# Estimates of Antibacterial Consumption in Timor-Leste Using Distribution Data and Variation in Municipality Usage Patterns

**DOI:** 10.3390/antibiotics10121468

**Published:** 2021-11-29

**Authors:** Lisa Harris, Alexander Bongers, Jennifer Yan, Joshua R Francis, Ian Marr, Susanna Lake, Santana Martins

**Affiliations:** 1 Procurelink, North Geelong, VIC 3215, Australia; alex@procurelink.io; 2 Menzies School of Health Research, Charles Darwin University, Casuarina, NT 0811, Australia; jennifer.yan@menzies.edu.au (J.Y.); josh.francis@menzies.edu.au (J.R.F.); dr.ian.marr@gmail.com (I.M.); 3 Murdoch Childrens Research Institute, Melbourne, VIC 3000, Australia; susanna.lake@mcri.edu.au; 4 Servico Autonomo Medicamentos e Equipamentos da Saude, Dili, Timor-Leste; santanamartins66@gmail.com

**Keywords:** antimicrobial consumption, antimicrobial usage, antimicrobial resistance, distribution data, low-middle income country, logistics management information system, Timor-Leste

## Abstract

The association between antimicrobial resistance and antimicrobial usage has become a growing global concern. Many lower-middle income countries including Timor-Leste (TL) have limited information on antimicrobial usage, although recent research suggests increasing resistance rates among human pathogens there. The aim of this study was to use distribution data to estimate antibiotic consumption at both the national and sub-national level in Timor-Leste, stratifying into resistance class and adherence to the national essential medicines list (EML) and WHO AWaRe guidelines. A retrospective review of distribution data from Timor-Leste central medical store (SAMES) was undertaken to give a defined daily dose (DDD)/1000 inhabitants/day using WHO methodology. National antibiotic distribution in the TL EML in 2019 was estimated at 11.1 DDD/1000 inhabitants/day, comparable to consumption rates observed in other lower-middle-income countries using similar methodology. Differences in distribution quantities were noted between municipalities, with 4 of the 13 municipalities notably above the national average and around 32% of listed restricted antimicrobials distributed incongruent with the EML. This study provides insights into estimated antimicrobial consumption in Timor-Leste that has previously been poorly defined. Estimates of consumption can be used to understand emerging resistance in this small island nation, add to the body of knowledge on antimicrobial use to advise policy and guideline development, and help with stewardship activities.

## 1. Introduction

Excess consumption and poor prescribing of antimicrobials is a known driver of antimicrobial resistance (AMR) [1] and is believed to be a serious threat to global public health [2,3]. Broader spectrum antibiotics, once reserved for more serious diseases are now being used more liberally in the context of emerging resistance [4]. Antimicrobial resistance is estimated to affect around 700,000 people every year and, if no urgent action is taken, could increase to 10 million every year by 2050, with the highest burden in Asia [5,6]. While AMR affects all countries, its impact is disproportionately higher in low- and-middle-income countries (LMIC) due to resource limitations and underdeveloped health infrastructure [7]. Timor-Leste, a small island LMIC in South East Asia, is particularly vulnerable to AMR due to low government spending on health care, limited or poorly implemented treatment guidelines, need for adequately trained medical staff, under resourced infrastructure, and cultural beliefs around antibiotic use [8]. These factors along with weak regulatory mechanisms promote inappropriate use of antimicrobials in Timor-Leste and other LMIC [7].

Antimicrobial stewardship (AMS) in combination with infection control has been shown to reduce antimicrobial resistance in hospitals, which in turn has shown a reduction in healthcare costs [9,10]. Understanding antimicrobial use and prescribing is a key component of antimicrobial stewardship. LMIC have unique challenges in implementing AMS strategies [11]. Data collection to inform antimicrobial consumption is essential to inform policies, regulations, and undertake interventions to improve the use of antimicrobials [12]. Reporting on antimicrobial consumption is an important element of AMS surveillance and provides important information to understand the association of antimicrobial use and resistance. Given that Asia is reported to bear the highest burden of antimicrobial resistance related disease, it is important to understand antimicrobial consumption within the Asia Pacific region. Understanding consumption data in countries that have limited or unknown reporting is needed to address poor antimicrobial prescribing and potential over use. Limited information exists around antimicrobial use and consumption in Timor-Leste, and this study hopes to address this issue.

The World Health Organization (WHO) defined daily dose (DDD) [13] is a globally accepted unit of measure for drug consumption to compare rates between countries, municipalities/regions, hospitals, and wards. The WHO Report on Surveillance of Antibiotic Consumption [12] states that “consumption data can be retrieved from a number of sources” including “procurement data by the wholesaler or sales data from wholesaler to health care facilities and pharmacies”; however, the closer the measure of consumption to the patient prescription, the more accurate a measure of the consumption rate. Distribution data can be used as an estimate when more comprehensive data are inadequate or not available.

LMICs often have a small procurement budget, where high rates of infectious disease-related mortality are disproportionate to spending on antimicrobials, often with low DDD/1000 inhabitants/day consumption. Servico Autonomo Medicamentos e Equipamentos da Saude (SAMES) is the central medical store in Timor-Leste and is the primary procurement agency for health facilities in Timor-Leste, including the national referral hospital (HNGV), five regional referral hospitals, and smaller district health services. Using SAMES procurement data from 2019, we aimed to estimate consumption rates of antimicrobials in Timor-Leste. Estimated consumption rates can be used to understand national antibiotic use, municipality variation, and overall consumption. Antimicrobial usage and prescribing patterns are recognized as drivers of antibiotic resistance. The outcome of this study has found evidence of higher consumption of antimicrobials in a number of municipalities and overuse of broader spectrum antibiotics outside of that outlined in policies and guidelines such as the Timor-Leste Essential Medicines List (EML) [14] and WHO Access, Watch, Reserve (AWaRe) guidelines [15].

## 2. Materials and Methods

### 2.1. Study Design and Period

A retrospective observational study of WHO classified antibacterial distribution data to 13 municipalities within Timor-Leste was conducted for the period 1 January 2019 to 31 December 2019.

Data were collected from distribution records stored on SAMES mSupply^®^ database. SAMES is the central medical store for public health facilities in Timor-Leste and sole distributor of medicines, equipment, and consumables.

There are around 65 community health centres and 198 health posts (with a plan to increase to >400), including a tertiary referral hospital, Hospital Nacional Guido Valadares (HNGV) in Dili, and 5 rural referral hospitals in Baucau, Maliana, Maubisse, Oecussi, and Suai [16].

Antimicrobials were categorised according to the WHO report on Surveillance of Antibiotic Consumption: 2016–2018 early implementation [12] and WHO Access, Watch, Reserve restriction classification system [15] and the latest edition of the Timor-Leste EML (2015) [14].

### 2.2. Data Analysis and Data Provenance

An observational period of one year was chosen to avoid seasonality and capture monthly to 3 monthly distribution cycles. Antimicrobial distribution data were captured from SAMES electronic logistics management information system (LMIS) (mSupply^®^), in units of medication as tablets, capsules, syrups, or vials and converted to mg of drug distributed. Data were extracted into Excel^®^ (Microsoft Corporation, Redmond, WA, USA) and further converted into a defined daily dose (DDD) through the anatomical therapeutic compound (ATC) classification system described by the WHO collaborating Centre for Drug Statistics Methodology (ATC/DDD, 2021).

### 2.3. Population

Census population data were used as the denominator to calculate DDD/1000 inhabitants/day [17]. All 13 municipalities, Aileu (population 44,325), Ainaro (population 59,175), Baucau (population 111,694), Bobonaro (population 92,049), Cova Lima (population 59,455), Dili (population 234,026), Ermera (population 117,064), Lautem (population 59,787), Liquica (population 63,403), Manatuto (population 42,742), Manufahi (population 48,628), Oecusse-Ambeno (population 64,025), and Viqueque (population 70,036) were included in the study giving a total population of 1,066,409 inhabitants studied.

### 2.4. Calculation of Estimated Antibiotic Consumption Rate

DDD/1000 inhabitants/day calculation was used to determine national and sub-national distribution and estimated consumption rates [13]. Total estimated consumption rates and comparisons between municipalities were reviewed and compared to recommended distribution of antimicrobials outlined in the Timor-Leste EML (2015). Compliant use of restricted antibacterials was achieved by examining restricted antimicrobials constant with the WHO AWaRe classification of restricted antimicrobial through distribution quantities sent to services outside of the tertiary referral or rural referral facilities who are able to access restricted antimicrobials as per the EML.

DDD used by each municipality was calculated using the following calculation:

Annual SAMES distribution mass of drug (mg)/WHO ATC DDD (mg) for each drug.

Consumption DDD was divided by the number of inhabitants, multiplied by 1000 to calculate a DDD/1000 inhabitant annually.

The daily consumption rate was calculated by dividing the DDD/1000 inhabitants annually by 365 days.

### 2.5. Antibiotic Inclusion Criteria

Antibacterials for systemic use (WHO ATC classification code J01) and nitroimidazole derivatives (WHO ATC classification code P01AB), were included as these make up the bulk of antimicrobial prescribing in most municipalities and globally. All other codes J02 (Antimycotics for systemic use), D01BA (Antifungals for systemic use), J05 (Antivirals for systemic use), J04A (Antimycobacterials for treatment of tuberculosis), and P01B (Antimalarials) were excluded.

Distribution data from SAMES for all agents listed above, available in the latest edition of the Timor-Leste National List of Medicines (EML 2015 [14]) were included in this study. Antibiotics that satisfied the inclusion criteria were evaluated for DDD/1000 inhabitants/day to determine estimation of consumption and listed below (Table 1).

### 2.6. Ethics Approval

Ethics approval was granted by the Instituto Nasional da Saude Research Ethics and Technical Committee in Timor-Leste and the Human Research and Ethics Committee of the Northern Territory Department of Health and Menzies School of Health Research in Australia.

## 3. Results

### 3.1. Antimicrobial Distribution in Timor-Leste

**Figure 1:**
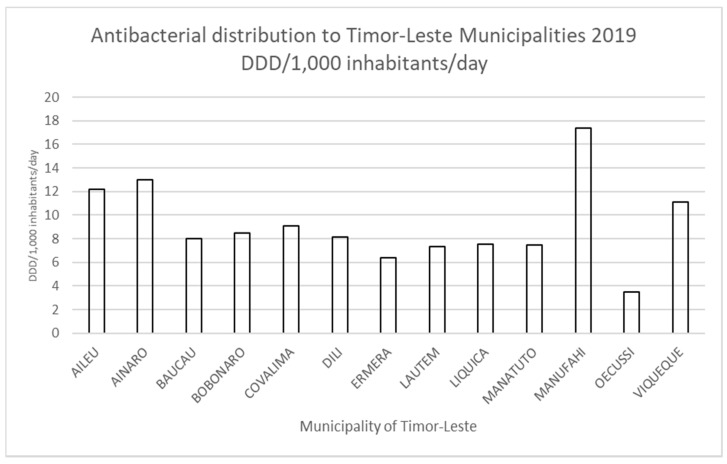
Antibacterial distribution volumes for municipalities of Timor-Leste 2019 DDD/1000 inhabitants/day.

The total volume of antibacterial medication distributed from SAMES to Timor-Leste’s 13 municipalities for 2019 was calculated at 4,320,962 DDDs, with the estimated total consumption rate of 11.1 DDD/1000 inhabitants/day. Consumption rates within the municipalities ranged from 3.5 DDD/1000 inhabitants/day (Oecussi municipality) to 17.4 DDD/1000 inhabitants/day (Manufahi municipality) with a mean DDD/1000 inhabitants/day of 8.1 (Figure 1).

**Figure 2:**
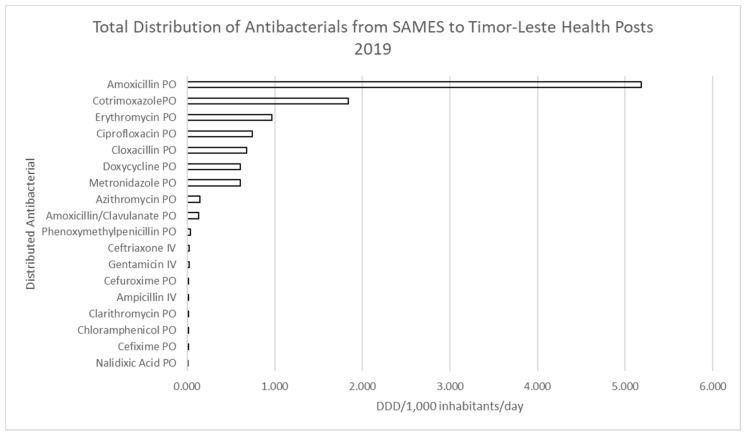
Distribution quantities of antibacterials for Timor-Leste 2019 DDD/1000 inhabitants/day.

**Figure 3:**
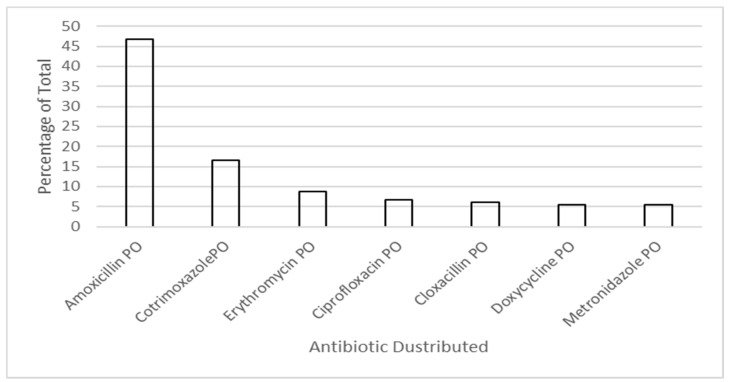
Percentage high distribution antibacterials for Timor-Leste 2019.

Eighteen antibacterials (99.8%) had a distribution rate greater than 0.01 DDD/1000 inhabitants/day and considered significant (Figure 2). Amoxicillin made up the majority of antibacterials distributed with an average of 5.2 DDD/1000 inhabitants/day, accounting for over 45% of all antibiotics distributed (Figure 3). Sulphamethoxazole+trimethoprim oral formulation (PO) (1.8 DDD/1000 inhabitants/day), Erythromycin PO (1 DDD/1000 inhabitants/day), ciprofloxacin PO (0.7 DDD/1000 inhabitants/day), cloxacillin PO (0.67 DDD/1000 inhabitants/day), doxycycline PO (~0.6 DDD/1000 inhabitants/day), and metronidazole PO (~0.6 DDD/1000 inhabitants/day) were amongst the most commonly prescribed (Figure 2).

Seven oral antibacterials (amoxicillin, Sulphamethoxazole+trimethoprim, erythromycin, ciprofloxacin, cloxacillin, doxycycline, and metronidazole) made up over 95% of distributed antibiotics by SAMES in 2019 to Timor-Leste health facilities (Figure 3).

Oral amoxicillin distribution sub-nationally accounted for 38 to 68% of total antibacterials distributed to each of the municipalities (Table 2). Around 70% of the municipalities studied were distributed high levels of amoxicillin over the national average of 45% of amoxicillin compared to other distributed antibacterials.

#### 3.1.1. Consumption of Restricted Items

Restricted antibacterials were defined from the Timor-Leste EML (2015) [14] which align with WHO AWaRe classification of restricted antimicrobials [15]. Restricted antimicrobials accounted for 94,971.58 DDDs, or 2.2% of total antimicrobial consumption. A total of 68.3% of restricted antimicrobials were used in hospital facilities, with the remainder (31.7%) used in non-hospital facilities.

#### 3.1.2. Comparison of Distribution and Dispensing Data

HNGV also uses mSupply^®^ as an LMIS for stock control and as dispensing software to patients. In 2019 SAMES recorded distributing 257,144 DDD’s of antibacterials defined in Table 1, to HNGV. HNGV recorded dispensing 227,957 DDD’s of the same antibacterials to both inpatient and outpatient departments, giving an estimated 89% of medications distributed in 2019 to HNGV, dispensed to patients at the tertiary referral hospital.

## 4. Discussion

This analysis represents the first known review of antimicrobial consumption in Timor-Leste and provides insights into antibacterial use and possible associations with increasing resistance. This study analyses data using the ATC/DDD methodology outlined by the WHO as a standard for calculating consumption rates. As Timor-Leste does not have another useful way of estimating consumption nationally due to limited IT infrastructure in most health facilities and pharmacies, SAMES distribution data appear to be appropriate surrogates of consumption using national public distribution data. This method gives an estimate of antibacterial consumption by municipality and usage of restricted antimicrobials by facility type (tertiary referral through to health post). This way of representing consumption may over-estimate antibacterial use due to wastage at the facility level, but it is the best available proxy source of data for true consumption.

We found that antibacterial consumption rates varied within the 13 municipalities surveyed across Timor-Leste and were observed to be heterogenous. The total distribution and estimated consumption rate of 11.1 DDD/1000 inhabitants/day is well within the range published by the WHO report on antimicrobial consumption within 65 countries [13] which estimated global consumption rates between 4.4 and 64.4 DDD/1000 inhabitants/day. Low consumption rates for LMIC have also been found by other studies [18] due to lower health spending and insufficient procurement budgets. Timor-Leste’s estimated antibacterial consumption of 11.1 DDD/1000 inhabitants per day is consistent with other LMIC such as Philippines, Indonesia, and India, using the same methodology, estimated between 5 and 15 DDD/1000 inhabitants/day [18].

Seven medications accounted for more than 95 percent of all antibacterials included in the observational study. The WHO report on antimicrobial consumption within 65 countries [13] found 45 medications made up all antibiotic consumption in the Western Pacific Region. Given that many of the antibacterials are closely related on this list, the WHO EML may be a more reliable indicator. The Timor-Leste EML [14] is estimated to be 84% compatible with the WHO EML [19]. A total of 68 of 239 medicines on the Timor-Leste EML are antimicrobials, of which 25 of these are antibacterials. Reliance on a small group of seven antibacterials to treat infectious diseases in Timor-Leste is lower than expected when comparing national guidelines and antimicrobials use with other LMIC [20] and raises the question around treatment efficacy and development of resistance to these pharmaceuticals. HNGV has a hospital guideline for empiric antibiotic use, with plans to develop a national empiric antibiotic guideline for the Ministry of Health (MOH). Previously, there were limited local data on causative pathogens of bacterial infection, and local antimicrobial susceptibility and resistance patterns to inform these empiric guidelines. Following recent investment in the diagnostic microbiology service at the national referral laboratory, there is now capacity to perform this testing and for the data to guide development of empiric guidelines and inform selection and procurement of an appropriate range of antibacterials for empiric and targeted use.

Amoxicillin was found to be the highest distributed antibacterial in 12 of the 13 municipalities. One municipality, Oecussi, reported no distribution of amoxicillin in 2019; however, there were 14,000 500mg tablets and 1800 bottles of dry powder for suspension (125 mg/5mL concentration, 100 mL bottles) distributed to Oecussi in 2018. Due to the large order of amoxicillin in 2018, Oecussi municipality did not order or were supplied amoxicillin in 2019. This significant year-to-year variability highlights the difficulty of using single year data to understand antimicrobial distribution patterns. It also raises questions about the ability to maintain recommended storage conditions in the tropical climate and the potential impact of prolonged storage on medication viability. Overuse of amoxicillin has the potential to drive resistance. Amoxicillin resistance in Enterobacteriaceae has been estimated from the national laboratory in Timor-Leste [21]. The latest published resistance rates from the national laboratory estimates *E. coli* resistance at over 90% for amoxicillin, which is higher than other countries reporting amoxicillin *E. coli* resistance rates [22,23]. More research is required to determine if this high amoxicillin use is a driver of reported resistance. Cotrimoxazole, erythromycin, and ciprofloxacin, together, contributed to around 30% of total systemic antibiotics distributed. The interim report on *E. coli* resistance from the national laboratory showed resistance rates of 79% in cotrimoxazole and 33% in ciprofloxacin. There is limited information around resistance in erythromycin. Due to the small number of isolates in this report, further research is required, however, macrolides and quinolones are known drivers of resistance and even these small numbers have highlighted the impact of overuse of these antibiotics.

The proportion of restricted antimicrobials prescribed outside of hospitals was high, despite guidance within the EML. The EML recommends that restricted antimicrobials be reserved for specialist use in hospitals. However, 31.70% of consumption for these antimicrobials occurred outside these facilities. Further information is required to understand these patterns and whether they are driven by antimicrobial prescribing, discrepancies in the EML mapping of facilities, upgrading of facilities where restricted antimicrobials may be used since the EML was published, or simply discrepancies in distribution. Antimicrobial susceptibility testing and resistance data are not commonly available outside of the national referral hospital HNGV in Dili, and thus antibiotic prescription in all other settings is on the basis of empiric and syndromic prescribing, rather than targeted use in the setting of known AMR.

The strength of this study is that SAMES is the sole public supplier of medication and keeps a large database for distribution of antimicrobials to Timor-Leste. These data currently are the best estimate of antimicrobial use in Timor-Leste. HNGV is the only facility in Timor-Leste that records electronic dispensing data. Comparison of distribution data to HNGV from SAMES with inpatient and outpatient dispensing records documented around 89 percent of distributed antibacterials dispensed to patients. This would suggest fairly good correlation between distribution and dispensing data, in the absence of national data on dispensing or individual patient use. Medication wastage of around 11 percent is in line with that reported from other countries [24,25].

Limitations of this study are that data extracted from the main government procurement agency, SAMES, do not include antibiotics available through the private sector. There are approximately 15 other agencies that procure medication privately in Timor-Leste including individual pharmacies and NGOs. Secondly, while distribution data are the best estimate of consumption data in Timor-Leste, they do not provide insight at the patient level, and they may over-estimate consumption in a number of ways; for example, medication held at the municipality warehouse may expire and be discarded, be removed from the system through other means, or be sent to another municipality and not make it to a patient. An electronic prescribing program such as mSupply^®^ used at the national referral hospital HNGV could provide more reliable and continuous collection of national prescribing data if rolled out to health facilities in the municipalities, however, this would require significant implementation support.

While health facilities continue to collect paper-based data, it will be difficult to use dispensing data to estimate consumption. Single year data have shown that remote municipalities such as Oecussi may request medication in high volumes in one year and not request medication again the following year. This creates concerns around storage conditions and likely high probability of large volumes of drug expiring and being discarded prior to the next delivery.

Longitudinal trend data obtained over multiple years are required to accurately analyse and interpret antibacterial distribution. Accessing drug destruction records would help understand the volume of expired stock. Drug destruction records were not observed in this study as they were not available at all municipalities and not routinely recorded.

This study has focused only on antibacterials. Future research investigating distribution data for all antimicrobials more generally in Timor-Leste and collecting trend data over a number of years would be useful to guide Timor-Leste’s policies for AMU and AMR. It would also provide accurate data for comparison as well as regional and global analysis. Other mechanisms to evaluate antimicrobial use, such as point prevalence surveys (PPS) are currently being analysed and awaiting publication. Data from these projects could be used to compare global consumption rates, prevalence of use and prescription appropriateness to inform national policies and guidelines and to understand antimicrobial resistance in Timor-Leste and sub-nationally in the municipalities.

## 5. Conclusions

This study has provided the first known antibacterial consumption data for Timor-Leste and has shown considerable variation within the municipalities. It highlights that comparison of country data alone may overlook important sub-national data variations. The small number of antibacterials used and high use of amoxicillin compared to other agents are of interest, particularly as laboratory capacity to detect clinical cases of bacterial infection and accumulate local antimicrobial susceptibility and resistance data builds. These data, on AMU and AMR, together will inform development of Timor-Leste’s first national empiric prescribing guidelines based on local country data. Ongoing AMU and AMR surveillance are crucial to continue advocating for access to appropriate antimicrobials while avoiding unnecessary or excessive antibiotic use.

## Figures and Tables

**Table 1:** Antibacterials for systemic use included in this study.

Antimicrobial	ATC Code	WHO ATC DDD	Route	Restriction
Amoxicillin	J01CA04	1.5 g	Oral	HP1
Amoxicillin/Clavulanate	J01CR02	1.5 g (amoxicillin)	Oral	DHC
Ampicillin	J01CA01	6 g	Injection	DHC
Azithromycin	J01FA10	0.3 g	Oral	DHC
Benzathine Benzylpenicillin	J01CE08	3.6 g (benzyl penicillin)	Injection	HP1
Benzyl Penicillin	J01CE01	3.6 g	Injection	HP1
Cefixime	J01DD08	0.4 g	Oral	DHC
Ceftriaxone	J01DD04	2 g	Injection	Spec
Cefuroxime	J01DC02	0.5 g	Oral	Spec
Chloramphenicol	J01BA01	0.3 g	Oral	HP1
Chloramphenicol	J01BA01	0.3 g	Injection	HP1
Ciprofloxacin	J01MA02	1 g	Oral	DHC
Clindamycin	J01FF01	1.8 g	Injection	Spec
Clindamycin	J01FF01	1.2 g	Oral	Spec
Cloxacillin	J01CF02	2 g	Injection	HP1
Cloxacillin	J01CF02	2 g	Oral	HP1
Cotrimoxazole 400 mg + 80 mg	J01EE01	4 tablets/40 mL	Oral	HP1
Doxycycline	J01AA02	0.1 g	Oral	HP1
Erythromycin	J01FA01	1 g	Oral	HP1
Gentamicin	J01GB03	0.24 g	Injection	SDHC
Meropenem	J01DH02	3 g	Injection	Spec
Metronidazole	P01AB01	2 g	Oral	HP1
Metronidazole	J01XD01	1.5 g	Injection	HP1
Nalidixic Acid	J01MB02	4 g	Oral	DHC
Phenoxymethylpenicillin	J01CE02	2 g	Oral	HP1
Vancomycin	J01XA01	2 g	Injection	Spec
Cefazolin	J01DB04	3 g	Injection	Hosp
Cefotaxime	J01DD01	4 g	Injection	Spec
Ceftazidime *	J01DD02	4 g	Injection	Spec
Clarithromycin	J01FA09	0.5 g	Oral	Hosp
Fusidic Acid	J01XC01	1.5 g	Oral	HNGV Spec

* Ceftazidime not on 2015 EML (special order), restricted antibiotic classification by Level of Use (LOU), (Spec = specialist restricted medicines to be used on specialist prescription from hospitals only; HNGV Spec = for use by respective specialists in the National hospital; Hosp = for use in National and District Referral hospitals; DHC = for use by District Health Centre and all hospitals; SDHC = for use by Sub-district Health Centres and all District Health Centres and all hospitals; HP1= for use by all health facilities).

**Table 2:** Amoxicillin distribution to municipalities in DDD/1000 inhabitants/day and percentage of antibacterial distributed to the municipalities included in the study.

Municipality	Total DDD/1000 Inhabitants/Day	DDD/1000 Inhabitants/Day Amoxicillin	% Amoxicillin (of Total Distributed)
Aileu	12.2	4.6	37.7
Ainaro	13	4.9	37.7
Baucau	8	4.1	51.2
Bobonaro	8.5	3.8	44.7
Cova Lima	9.1	5.3	58.2
Dili	8.1	4.5	55.5
Ermera	6.4	2.5	39.1
Lautem	7.3	4.5	61.6
Liquica	7.5	3.5	46.6
Manatuto	7.5	5.1	68
Manufahi	17.4	6.8	39.1
Oecusse-Ambeno	3.5	0	0
Viqueque	11.1	4.9	44.1

## Data Availability

The data for this study are available from the corresponding author on request.
